# Self-management activation for low back pain and its influencing factors among intensive care unit nurses: a multicenter cross-sectional study

**DOI:** 10.3389/fpubh.2025.1665408

**Published:** 2025-10-01

**Authors:** Zhi Zeng, Li Wan, Xiuru Yang, Fenglin Yan, Zhenghua Liang, Mei He

**Affiliations:** Mianyang Central Hospital, Mianyang, China

**Keywords:** nurse, low back pain, self-management, occupational health, ICU

## Abstract

**Objective:**

To investigate the current status of self-management activation of low back pain (LBP) among intensive care unit (ICU) nurses and analyze the influencing factors, to provide a reference for intervention strategies to improve their self-management activation of LBP.

**Methods:**

Through a cross-sectional research method, 366 ICU nurses from five tertiary-level hospitals in Mianyang City were selected in January–March 2025 using a convenience sampling method. With ternary interaction determinism as the theoretical basis, the general information questionnaire, the Participants Activation for self-management of Back Pain (PAMQ), the presenteeism behavior scale, and the perceived social support scale (PSSS) scale were used to conduct the survey. Descriptive statistics, univariate analysis, and multiple linear regression analysis were employed to describe the current status of self-management activation for LBP among ICU nurses and to identify its associated factors.

**Results:**

ICU nurses scored (37.93 ± 5.69) on the PAMQ with a score of 69.0%, and the related self-management awareness, self-management beliefs, and self-management knowledge dimensions scored in the order of sub 75.1, 68.2, and 66.9%. Correlation analysis revealed that the self-management activation for LBP among ICU nurses was negatively correlated with presenteeism (*p* < 0.001) and positively correlated with perceived social support (*p* < 0.01). Multiple linear regression analysis showed that age, years of work experience, educational level, frequency of exercise, participation in LBP prevention training, presenteeism, and perceived social support were significantly associated with self-management activation for LBP among ICU nurses (*p* < 0.05), collectively explaining 63.6% of the total variance.

**Conclusion:**

The overall self-management activation for LBP among ICU nurses needs to be improved urgently. Although these nurses demonstrate a strong motivation for self-management, they possess insufficient knowledge regarding LBP. Therefore, future interventions should be tailored to key factors such as ICU nurses’ age, years of work experience, education level, exercise frequency, participation in LBP prevention training, presenteeism, and perceived social support. Developing such precise and systematic intervention strategies will enhance self-management activation for LBP, reduce the incidence of LBP, and ultimately promote the wellbeing of the nursing workforce.

## Introduction

Low Back Pain (LBP) refers to symptoms of pain, numbness, and/or limited mobility in the lower back, resulting from prolonged work-related strain, after excluding other potential causes based on medical evaluation (1). The global burden of LBP is primarily attributed to three modifiable risk factors: occupational ergonomics, smoking, and elevated Body Mass Index (BMI), among which occupational ergonomics is the most significant contributor (2). Nurses are at an elevated risk of LBP compared to other professions (3). Surveys have shown that the prevalence of occupational low back pain among nurses ranges from 50 to 80% (4). The results of a multicentre study in China showed that the prevalence of LBP among nurses was 91.0%, much higher than that reported in other countries (5). Notably, nurses working in intensive care units (ICUs) exhibited higher prevalence and frequency of LBP compared to nurses working in general wards, owing to their long-term fixed posture, frequent carrying activities, and continuous standing (6, 7). A meta-analysis revealed that the pooled prevalence of LBP among ICU nurses was 76.0%, significantly higher than the prevalence of 66.9% observed in general ward nurses. Furthermore, LBP in ICU nurses demonstrates a trend of high incidence and a younger age of onset (8). LBP is widespread among ICU nurses, creating a dual threat. It undermines the stability of the nursing staff and, by compromising care quality, ultimately endangers patient safety. Thus, systematically analyzing and effectively addressing this issue is essential to safeguarding the integrity of the nursing profession and the quality of healthcare.

As a chronic condition with long-term and recurrent attacks, LBP is difficult to cure using only short-term ergonomic, exercise, or psychological interventions (9). Thus, ICU nurses affected by LBP should undertake sustained self-management practices. Self-management activation is a comprehensive reflection of patients’ knowledge, skills, and confidence in disease management, which reflects their self-health management behaviors and can be used as an indicator to predict health behaviors and outcomes (10). For ICU nurses, a higher level of self-management activation for LBP can help them maintain management behaviors, thereby effectively relieving pain, reducing the risk of recurrence, promoting functional recovery, and ultimately improving their work and quality of life. However, most of the studies focus on the prevalence and influencing factors of LBP among ICU nurses, while research on their self-management activation for LBP is relatively lacking (11). Furthermore, the specific factors that influence the self-management activity of LBP among ICU nurses require further investigation.

Ternary interaction determinism states that environmental factors, personal factors, and behavioral responses are distinct yet interrelated, with behavioral responses resulting from the interaction of internal and external factors (12). In ICU nurses, both personal and environmental factors affect their behavioral responses during LBP self-management, namely, the activation of self-management. Previous studies have shown that presenteeism is closely related to nurses’ LBP (13). Presenteeism refers to the behavior of individuals continuing to work despite poor health (14). In the high-intensity work environment of the ICU, nurses’ presenteeism is even more pronounced (15). However, the relationship between presenteeism and self-management activation levels for LBP among ICU nurses remains unexplored. Therefore, it is necessary to consider this factor when exploring the self-management activation of LBP among ICU nurses. Moreover, perceived social support refers to an individual’s subjectiv perception of objective support, and is characterized by a positive emotional experience stemming from the feeling of being supported (16). Studies suggest that robust social support can bolster patients’ confidence in treatment and management, thereby encouraging active coping with the disease and the adoption of self-management behaviors (12). Based on these considerations, this study used ternary interaction determinism as a theoretical analysis framework, with the self-management activity level of low back pain as the dependent variable, and individual factors (presenteeism) and environmental factors (perceived social support) that may influence it as independent variables. Further, the study explored the current situation and factors influencing self-management activation in ICU nurses, providing a reference for formulating health promotion programs specifically designed for self-management activation in this population.

## Methods

### Study design

This multicentre cross-sectional study, conducted from January 2025 to March 2025, was approved by the Ethics Committee of Mianyang Central Hospital (registration number: S202403139-01).

### Participants

A convenience sampling method was adopted to select registered ICU nurses from five tertiary hospitals in Mianyang City between January and March 2025. Inclusion criteria included (1) employed as an ICU nurse; (2) having worked as an ICU nurse for at least 1 year; (3) experiencing symptoms such as pain, discomfort, and limitation of movement in the low back within the past year; and (4) informed consent and willingness to participate in this investigation. The exclusion criteria were (1) history of lumbar trauma or surgery; (2) presence of pathological LBP caused by tumors, ankylosing spondylitis, etc.; (3) physiological LBP caused by menstruation, pregnancy, or breastfeeding; (4) ICU nurses for further training, rotation, and internship; (5) Nurses in emergency ICU, pediatric ICU and ICU of various specialties.

An *a priori* power analysis was conducted using G*Power 3.1 to determine the required sample size for this study (17). The analysis was based on a multiple linear regression model with 19 predictors, aiming to detect a medium effect size (Cohen’s *f*^2^ = 0.15) with a significance level (*α*) of 0.05 and a statistical power of 0.90. The results indicated that a minimum sample of 221 participants was required. To account for an anticipated attrition rate of 20%, we planned to recruit a total of 277 participants, thereby ensuring adequate statistical power for the analyses.

### Instruments

#### Demographic questionnaire

The demographic variables were as follows: (1) general demographic characteristics, including age, gender, BMI, education level, marital status, etc.; (2) work-related information, including working years, frequency of night shifts, frequency of exercise, and frequency of bending and heavy lifting, etc.; and (3) LBP-related information, including LBP protection training experience, duration of LBP, number of visits for LBP in the previous 2 months, and maximum pain intensity experienced. Pain levels were assessed using the NRS numeric pain intensity assessment scale, a 0–10 scale indicating no pain to the most pain, with 0–3 as no or mild pain, 4–6 as moderate pain, and 7–10 as severe pain.

#### Participants’ activation for self-management of back pain

This scale was developed by Nktata et al. (18). The Chinese version of the PAMQ was used to assess the self-management activation level of nurses (19). The questionnaire comprises 11 items across three dimensions: self-management beliefs, self-management awareness, and self-management knowledge. All items were scored on a 5-point Likert scale (1 = completely disagree and 5 = completely agree), with a total score of 11–55. Higher scores indicate a higher activation level for nurses’ self-management of LBP. The Cronbach’s *α* coefficient of the scale was 0.821. Based on previous relevant literature (20), the calculation method for the PAMQ score rate was defined as follows: (actual score/maximum possible score) × 100%. A score rate greater than 85% was classified as a high level, less than 60% as a low level, and between 60 and 85% as a moderate level.

#### Presenteeism behavior scale

The scale was developed by Lu et al. to measure clinical nurses’ presenteeism behavior (21), using items such as ‘You compel yourself to attend work despite feeling sick’ and ‘You compel yourself to attend work despite physical symptoms such as headache or backache’. Participants were asked to recall and rate the number of times they had the given behavior in the past 6 months, scoring 1 for ‘never’, 2 for ‘1 time’, 3 for ‘2–5 times’, and 4 for ‘more than 5 times’. The average score of the two items was taken as the presenteeism score. The higher the score, the higher the frequency of presenteeism. The Cronbach’s *α* coefficient of the scale was 0.84.

#### Perceived social support scale (PSSS)

The PSSS is mainly used to assess individuals’ perceived social support (22). It was translated and revised by Zhong et al. (23), and includes three dimensions of family, friend, and other supports, with a total of 12 items. All items were scored on a 7-point Likert scale (1 = strongly disagree, 7 = strongly agree), with a total score of 11–55. The higher the score, the more social support the individual feels. The Cronbach’s *α* coefficient for this scale was 0.894.

### Data collection

This study employed a mixed-methods approach for online and offline data collection. Online data collection involved contacting the ICU nurse managers of various hospitals via telephone or WeChat to explain the study’s purpose and content and obtain their consent. Subsequently, ICU nurse managers distributed the QR code for the electronic questionnaire to ICU nurses. The questionnaire included a unified set of instructions on the first page and an informed consent option; those who did not consent were exited from the survey, whereas those who consented could proceed by answering the questions. To avoid duplicate or missing data, each IP address was limited to one submission, and all questions were marked as ‘required’, with the questionnaire only being submitted upon completion of all fields. The researcher reviewed the questionnaires, excluding those with an approximate response time of 180 s or those where all questions were answered with the same option. The researcher collected offline data and personally administered the questionnaire to participants who met the inclusion criteria. Before the survey, the researcher elucidated the purpose and significance of the study. The researcher was present throughout the questionnaire completion process to address any enquiries from the participants. After collection, the researcher performed an initial review to ensure logical consistency and excluded any questionnaires that exhibited inconsistencies. Questionnaires were also examined for errors or omissions. The participants rectified any identifiers under the supervision of the researcher.

### Data analysis

Data were cross-verified through dual entry and analyzed using SPSS 27.0. Continuous variables were characterized by mean ± standard deviation, whereas categorical variables were summarized using frequencies, proportions, and percentages. Univariate analyses were conducted using independent-sample *t*-tests or one-way analysis of variance (ANOVA) as appropriate. The strength and direction of the linear relationships between pairs of continuous variables were determined using Pearson’s product–moment correlation coefficient. Multiple linear regression analysis was performed to identify significant predictors of the PAMQ. Statistical significance was set at *p* < 0.05.

## Results

A total of 400 questionnaires were distributed, of which 393 were returned, resulting in a response rate of 98.3%. After excluding invalid responses, 366 valid questionnaires were used for the analysis, yielding an effective response rate of 91.5%.

### PAMQ scores among ICU nurses

The PAMQ scores of the 366 ICU nurses were moderate. Table 1 presents the scores for each dimension and the total scores.

**Table 1:** PAMQ scores of ICU nurses (*n* = 366).

Variables	Score	Score ( $\bar{x}\pm s$ )	Score rate (%)
Total score of PAMQ	11–55	37.93 ± 5.69	69.0
Self-management consciousness score	2–10	7.51 ± 1.48	75.1
Self-management belief score	5–25	17.05 ± 2.92	68.2
Self-management knowledge score	4–20	13.37 ± 2.42	66.9

Score rate = (Actual score/Theoretical maximum score) × 100%.

### Univariate analysis of general characteristics and PAMQ scores among ICU nurses

The results of the univariate analysis indicated that there were statistically significant differences in PAMQ scores among ICU nurses in terms of age, gender, years of work experience, educational level, frequency of night shifts, frequency of bending and lifting heavy objects, exercise frequency, participation in LBP prevention training, duration of LBP, number of LBP-related medical visits in the past 2 months, and severity of LBP (all *p* < 0.05). However, no statistically significant differences in PAMQ scores were observed regarding BMI and marital status (*p* > 0.05). The specific data are presented in Table 2.

**Table 2:** General characteristics of ICU nurses and univariate analysis of their PAMQ scores (*n* = 366).

Variables	Item	Number (%)	Score ( $\bar{x}\pm s$ )	*F*/*t*	*p*
Age				4.468	0.012
	≤30 years old	162 (44.3%)	37.60 ± 5.81		
	31–40 years old	183 (50.0%)	37.82 ± 5.72		
	>40 years old	21 (5.7%)	41.48 ± 2.84		
Gender				−2.066	0.04
	Male	60 (16.4%)	36.55 ± 6.64		
	Female	306 (83.6%)	38.20 ± 5.46		
BMI				1.543	0.203
	<18.5	22 (6.0%)	38.68 ± 3.60		
	18.5–23.9	253 (69.1%)	38.23 ± 5.67		
	24.0–27.9	86 (23.5%)	37.02 ± 6.22		
	≥28.0	5 (1.4%)	35.00 ± 0.00		
Educational level				8.383	<0.001
	College and below	48 (13.1%)	34.92 ± 6.57		
	Bachelor’s degree	285 (77.9%)	38.30 ± 5.49		
	Master’s degree and above	33 (9.0%)	39.12 ± 4.72		
Marital status				−1.396	0.164
	Unmarried	149 (40.7%)	37.41 ± 6.44		
	Married	217 (59.3%)	38.29 ± 5.10		
Years of work experience				2.848	0.037
	≤5 year	103 (28.1%)	36.86 ± 6.28		
	6–10 year	106 (29.0%)	38.17 ± 6.01		
	10–15 year	129 (35.2%)	38.09 ± 5.18		
	>15 year	28 (7.7%)	40.21 ± 3.32		
Frequency of night shifts				6.186	<0.001
	0 times/month	34 (9.3%)	35.59 ± 5.49		
	1–5 times/month	56 (15.3%)	40.52 ± 4.64		
	6–10 times/month	134 (36.6%)	37.70 ± 4.93		
	>10 times/month	142 (38.8%)	37.69 ± 6.44		
Frequency of bending and lifting heavy objects				3.686	0.012
	<5 times/day	38 (10.4%)	39.47 ± 3.40		
	5–10 times/day	192 (52.5%)	37.14 ± 5.77		
	10–15 times/day	60 (16.4%)	39.45 ± 5.76		
	>15 times/day	76 (20.8%)	37.97 ± 6.04		
Frequency of exercise				13.873	<0.001
	0 times/week	176 (48.1%)	36.36 ± 5.41		
	1–3 times/day	170 (46.4%)	39.39 ± 5.56		
	>3 times/day	20 (5.5%)	39.40 ± 5.86		
Participation in LBP prevention training				107.53	<0.001
	No	244 (66.7%)	36.01 ± 5.60		
	Yes	122 (33.3%)	41.77 ± 3.50		
Duration of LBP				11.372	<0.001
	<1 year	138 (37.7%)	36.17 ± 6.17		
	1–3 year	126 (34.4%)	38.84 ± 5.25		
	>3 year	102 (27.9%)	39.20 ± 4.95		
Number of LBP-related medical visits in the past 2 months				5.81	0.003
	0 times	130 (35.5%)	38.29 ± 5.79		
	1–2 times	171 (46.7%)	38.47 ± 4.67		
	3–4 times	65 (17.8%)	35.78 ± 7.33		
Severity of LBP				9.18	<0.001
	Mild pain	164 (44.8%)	38.48 ± 4.89		
	Moderate pain	197 (53.8%)	37.74 ± 5.91		
	Severe pain	5 (1.4%)	27.8 ± 11.34		

### Analysis of scores and correlations among the PAMQ, presenteeism behavior scale, and PSSS in ICU nurses

The mean PAMQ score for ICU nurses was 37.93 ± 5.69. The mean presenteeism behavior scale score was 4.60 ± 2.19, and the mean PSSS score was 57.39 ± 7.77. The PAMQ scores were negatively correlated with the presenteeism behavior scale scores (*p* < 0.001) and positively correlated with the PSSS scores (*p* < 0.01). The specific data are presented in Table 3.

**Table 3:** Correlation analysis of PAMQ, presenteeism behavior scale, and PSSS in ICU nurses (*r*; *n* = 366).

Variables	PSSS	Presenteeism behavior scale
PAMQ	0.694**	−0.606**

**p < 0.001.

### Results of multivariate analysis of PAMQ scores in ICU nurses

Multiple linear regression analysis was conducted, with the PAMQ score of ICU nurses as the dependent variable, and the statistically significant variables from the univariate analysis, along with the scores of the Attendance Behaviorism Scale and the PSSS, as the independent variables. The results indicated that age, educational level, years of work experience, frequency of exercise, participation in LBP prevention training, presenteeism, and PSSS score were significantly associated with the self-management activation of LBP among ICU nurses, collectively explaining 63.6% of the total variance. The specific data are presented in Table 4.

**Table 4:** Results of multivariate linear regression analysis for PAMQ in ICU nurses (*n* = 366).

Item	*b*	*SE–b*	*Beta*	*t*	*p*
Constant	12.198	3.425		3.561	<0.001
Age	−2.563	0.568	−0.267	−4.515	<0.001
Educational level	1.14	0.419	0.094	2.718	0.007
Years of work experience	1.843	0.349	0.306	5.284	<0.001
Frequency of exercise	1.808	0.325	0.189	5.564	<0.001
Participation in LBP prevention training	2.969	0.411	0.246	7.216	<0.001
PSSS	0.34	0.034	0.464	10.001	<0.001
Presenteeism behavior scale	−0.511	0.12	−0.197	−4.261	<0.001

*b* is the unstandardized regression coefficient; *SE-b* is the standard error; *Beta* is the standardized regression coefficient. PSSS is the perceived social support scale. *R*^2^ = 0.650; adjusted *R*^2^ = 0.636.

## Discussion

### The self-management activation level of ICU nurses with LBP requires improvement

Studies have shown that LBP is a significant occupational health issue affecting nurses. It impairs nurses’ physical health and negatively impacts their work efficiency and quality of care. Furthermore, LBP can reduce job satisfaction, leading to burnout and intention to leave the profession (24). Therefore, there is an urgent need to improve the self-management ability of ICU nurses to promote their physical and mental health, ensure quality of care, and maintain the stability of the nursing workforce.

The results of this study indicate that the average score rate for the PAMQ among ICU nurses was 69.0%. The scores for the related self-management awareness, self-management beliefs, and self-management knowledge dimensions were 75.1, 68.2, and 66.9%, respectively, which needed to be improved. This is consistent with the findings of Zhang et al. (25), indicating that while these nurses are aware of LBP self-management, they lack sufficient knowledge reserves, skills, and confidence in coping with LBP. Several factors may contribute to this observation: (1) Individual Level: nurses’ lack of awareness of LBP hazards, lack of self-management knowledge, and weak management belief were the internal factors of their low self-management activation. (2) Organizational Level: The low self-management activity of nurses is externally influenced by factors such as the insufficient provision of patient-handling equipment in ward areas, coupled with a heavy workload (26), and a lack of organizational policies and training programs focused on preventing LBP. Relevant studies have indicated that nurses’ low awareness of LBP prevention, their level of knowledge, and whether they have received LBP prevention training are closely related to the incidence of LBP (27). It is recommended that nursing managers prioritize the implementation of LBP self-management education, prevention training courses, and corresponding assessments. This will enable nurses to correctly understand LBP, cultivate a positive awareness and belief in managing LBP, enhance their ability to cope with LBP, thereby preventing and alleviating the occurrence and progression of LBP.

### The self-management activation of LBP among ICU nurses is closely associated with a variety of factors

According to the theory of triadic reciprocal determinism, individual behavior arises from the interaction between personal factors and the external environment. Drawing on this theoretical framework, the study explores the factors influencing the self-management activation of LBP among ICU nurses. The findings indicate that the self-management activation of ICU nurses is significantly correlated with both intrinsic and extrinsic factors. These findings are further elaborated below.

### Personal factors associated with the self-management activation of LBP among ICU nurses

Significant associations were observed between ICU nurses’ PAMQ scores and personal factors, including age, years of work experience, education level, exercise frequency, and presenteeism. (1) Older ICU nurses demonstrated lower levels of self-management activation for LBP, potentially due to the increased risk of chronic or exacerbated symptoms associated with advancing age. The persistent experience of LBP may deplete physical resources and erode self-efficacy and hope, thereby reducing proactive engagement in self-management behaviors (28). (2) ICU nurses with more years of work experience demonstrate higher levels of self-management activation for LBP. This may be because experienced nurses typically have a stronger theoretical foundation and more advanced clinical skills. These strengths allow them to effectively access medical resources and social support networks, which in turn enables them to adopt more proactive and effective strategies when managing their LBP (29). Therefore, it is recommended that nursing managers establish a sharing platform to encourage experienced nurses to provide demonstrations and support in LBP self-management for older nurses. This approach facilitates precise interventions tailored to different groups, thereby enhancing the overall level of proactive engagement in LBP self-management across the team. (3) ICU nurses with higher educational levels demonstrate higher levels of self-management activation for LBP, which aligns with the findings of Al Sayah et al. (30). This may be attributed to the fact that individuals with higher education levels possess greater health information literacy, enhanced disease awareness and acceptance, a stronger capacity to learn, receive, and apply knowledge, better mastery of self-care skills, and a greater tendency to actively seek diverse sources of disease-related information. These attributes enable them to efficiently acquire knowledge related to self-health management and fully engage their potential in participating in disease management (31). (4) ICU nurses who engage in regular exercise exhibit higher levels of self-management activation for LBP. Exercise serves as a key measure for both the treatment and prevention of LBP, as it enhances spinal stability, alleviates pain, and improves quality of life (32). Additionally, exercise helps nurses alleviate work-related stress, improve negative emotions, and adopt a more proactive approach to managing LBP (33). Therefore, it is recommended that organizations provide professional, systematic exercise guidance for ICU nurses to enhance their participation in physical activity, thereby boosting their confidence and initiative in LBP self-management. (5) ICU nurses with higher scores in presenteeism exhibit lower levels of self-management activation for LBP. This is because nurses who work while ill often expend additional physical, psychological, and emotional resources. Regardless of whether their work is completed effectively, this process consumes substantial energy reserves (34). The emergence of presenteeism among ICU nurses can be attributed to two main factors. On one hand, traditional Chinese culture and professional ethics play a significant role. Nursing education in China has long emphasized the spirit of selfless dedication and professional commitment. Driven by emotional and moral factors, nurses tend to prioritize their work responsibilities over personal health (35). This view is also supported by the research of Gholian-Aval et al. (36). On the other hand, heavy workloads are a primary cause of presenteeism, which is fundamentally rooted in the relative shortage of nursing human resources. Therefore, it is recommended that nursing managers enhance health education for nurses, helping them to recognize the harms of presenteeism and challenge the traditional notion that “working while ill is a sign of dedication,” and they should be encouraged to rest and seek medical attention promptly when unwell. Additionally, efforts should be made to optimize the allocation of nursing human resources, adjusting staffing based on the specific demands and workload of the ICU, to reduce nurses’ work burden.

### External environmental factors associated with the self-management activation of LBP among ICU nurses

Environmental factors, including participation in LBP prevention training and perceived social support, were significantly correlated with ICU nurses’ self-management activation for LBP. (1) ICU nurses who have received training in LBP prevention exhibit higher levels of self-management activation for LBP, a finding consistent with Delshad et al. (37). This suggests that nursing managers should actively foster a culture of occupational safety, provide relevant LBP self-management training, correct misconceptions, and assist nurses in selecting appropriate coping strategies, thereby enhancing their self-management activation and promoting positive health behavior changes. (2) Social support, defined as the emotional, informational, or instrumental assistance from personal networks such as family and friends, is crucial for individuals in coping with challenges (38). Our study found that the higher the level of perceived social support among ICU nurses, the stronger their self-management abilities for LBP. The underlying reason is that support from family, hospitals, or the community can effectively buffer the emotional exhaustion nurses experience due to poor outcomes in LBP self-management (39). Concurrently, this sustained support can also enhance their confidence in managing their condition (i.e., coping self-efficacy), creating a positive psychological feedback loop that, in turn, encourages them to adopt self-management strategies more frequently (40). Based on these findings, nursing managers are encouraged to enhance support for ICU nurses by fostering a supportive organizational environment. This can be achieved through three key types of support: emotional comfort (e.g., providing mental health seminars), informational support (e.g., delivering training on low back pain prevention and management), and instrumental support (e.g., improving ergonomic equipment). Such comprehensive support is expected to increase self-management activation for LBP among ICU nurses, thereby promoting more effective self-management behaviors.

## Limitations

This study has several limitations. First, due to time and financial constraints, the investigation was limited to five tertiary hospitals in Mianyang, China, which restricts the representativeness of the sample. Future research should broaden the scope of investigation to improve the generalizability of the findings. Second, the cross-sectional design reveals only correlations among variables and cannot establish causal relationships. Future studies could incorporate qualitative interviews to explore underlying mechanisms in greater depth or employ longitudinal designs to validate causal links between variables. Finally, the study relied primarily on self-reported data, which may introduce recall bias and compromise the precision of the results. Subsequent research should integrate objective measurement indicators for validation.

## Conclusion

Gaining a thorough understanding of the level of self-management activation for LBP among ICU nurses and its related factors is of great practical significance for exploring the impact of LBP on nurses’ health management behaviors and psychological wellbeing. Guided by the triadic interaction theory, this study analyzed the current status and influencing factors of self-management activation for LBP among ICU nurses. The findings indicate that the self-management activation for LBP among ICU nurses requires further improvement. Age, years of work experience, educational level, frequency of exercise, participation in LBP prevention, presenteeism, and perceived social support are all closely associated with the activation of LBP self-management among ICU nurses. Nursing managers should develop targeted interventions based on the diverse characteristics of ICU nurses to promote their proactive engagement in LBP self-management, thereby preventing the onset and progression of LBP and enhancing occupational health.

## Data Availability

The original contributions presented in the study are included in the article/supplementary material, further inquiries can be directed to the corresponding author.
